# Accuracy and precision of variance components in occupational posture recordings: a simulation study of different data collection strategies

**DOI:** 10.1186/1471-2288-12-58

**Published:** 2012-06-18

**Authors:** Per Liv, Svend Erik Mathiassen, Susanne Wulff Svendsen

**Affiliations:** 1 Centre for Musculoskeletal Research, University of Gävle, Gävle, Sweden; 2 Occupational & Environmental Medicine, Department of Public Health and Clinical Medicine, Umeå University, Umeå, Sweden; 3 Danish Ramazzini Centre, Department of Occupational Medicine, Herning Hospital, Herning, Denmark

## Abstract

**Background:**

Information on exposure variability, expressed as exposure variance components, is of vital use in occupational epidemiology, including informed risk control and efficient study design. While accurate and precise estimates of the variance components are desirable in such cases, very little research has been devoted to understanding the performance of data sampling strategies designed specifically to determine the size and structure of exposure variability. The aim of this study was to investigate the accuracy and precision of estimators of between-subjects, between-days and within-day variance components obtained by sampling strategies differing with respect to number of subjects, total sampling time per subject, number of days per subject and the size of individual sampling periods.

**Methods:**

Minute-by-minute values of *average elevation*, *percentage time above 90°* and *percentage time below 15°* were calculated in a data set consisting of measurements of right upper arm elevation during four full shifts from each of 23 car mechanics. Based on this parent data, bootstrapping was used to simulate sampling with 80 different combinations of the number of subjects (10, 20), total sampling time per subject (60, 120, 240, 480 minutes), number of days per subject (2, 4), and size of sampling periods (blocks) within days (1, 15, 60, 240 minutes). Accuracy (absence of bias) and precision (prediction intervals) of the variance component estimators were assessed for each simulated sampling strategy.

**Results:**

Sampling in small blocks within days resulted in essentially unbiased variance components. For a specific total sampling time per subject, and in particular if this time was small, increasing the block size resulted in an increasing bias, primarily of the between-days and the within-days variance components. Prediction intervals were in general wide, and even more so at larger block sizes. Distributing sampling time across more days gave in general more precise variance component estimates, but also reduced accuracy in some cases.

**Conclusions:**

Variance components estimated from small samples of exposure data within working days may be both inaccurate and imprecise, in particular if sampling is laid out in large consecutive time blocks. In order to estimate variance components with a satisfying accuracy and precision, for instance for arriving at trustworthy power calculations in a planned intervention study, larger samples of data will be required than for estimating an exposure mean value with a corresponding certainty.

## Background

In occupational studies, increasing attention is paid to understanding exposure variability expressed as variance components, both for the purpose of focusing surveillance and intervention on appropriate targets [1,2] and in order to design efficient exposure assessment strategies for epidemiologic studies and intervention research [3-5]. In the context of biomechanical exposures, Mathiassen et al. [6,7] have proposed that variance components can be used as measures of physical variation in a task, job or occupation, and thus meet the need for variables describing this essential aspect in assessments of risks of developing musculoskeletal disorders. Variance components are derived from statistical random effects models [8], by which the total variance in data is partitioned into estimated variance components associated with different random factors, i.e. sources of variability, in the model. In occupational exposure studies, typical random factors are subjects and working days, and the corresponding variance components are referred to as the between-subjects variance and the between-days (within-subject) variance [1]. Fitting random effects models to exposure data has mainly been practiced in chemical exposure assessment [1,9-11], but a number of studies have used such models for biomechanical exposures as well [3,4,6,12-14]. Since variance components can be the primary exposure or outcome measure of a study, rather than just a tool for obtaining another variable of interest (for instance the precision of an estimated mean exposure), the issue of accuracy (absence of bias) and precision of different sampling strategies for determining variance components needs to be addressed. Few previous studies have been devoted to this issue. Mathiassen et al. [4] used bootstrapping to construct confidence intervals for the between-subjects variance, between-days variance and residual (within-day) variance in electromyography (EMG) data from cyclic assembly work. In another study, Mathiassen et al. [6] used theoretical formulas to construct confidence intervals for variance components of EMG measurements from subjects performing a constrained work task. These studies pointed to a considerable imprecision of the estimated variance components, in particular when exposure samples were few and short. For occupational posture recordings, which are the focus of this paper, it is common to collect data for only smaller parts of a working day (e.g. [15-18]) even if continuous recordings of a full work shift do occur (e.g. [19-23]).

Standard random effects models assume that the modelled random effects are uncorrelated. Probably, this assumption is often violated in occupational settings since exposures close in time tend to be more similar than exposures far apart in time [24,25]. In a study of upper arm postures in three occupations, Liv et al. [14] showed that the assumption of independent errors was less severely violated if a data sample was distributed among several short time periods during a shift than if the sample comprised fewer or only one longer time period. Due to autocorrelation, the observed variance of a mean exposure estimate was also larger than expected from theoretical formulae based on the posture variance components. Similar effects of autocorrelation can also be expected for sample estimates of variance components based on standard random effects models [14]. To our knowledge, this issue has not been investigated in the occupational exposure literature, and so the accuracy and precision of variance components derived by standard procedures under different data collection scenarios are only superficially understood.

The aim of this study was to investigate and discuss the accuracy and precision of estimators of variance components for upper arm elevation when data are collected using different sampling strategies, and to suggest and apply a bootstrap approach for investigating sampling performance in this context.

## Methods

The data used in this study were collected for an epidemiologic study of the relationship between upper arm elevation and shoulder disorders among house painters, car mechanics and machinists [21,26,27]. We chose to limit the present study to data from the car mechanics in order to limit the results of the study to manageable amounts. The data set was intended to consist of measurements of right upper arm elevation for five full shifts in each of 25 car mechanics. Posture data were collected by means of the Abduflex equipment [28], which at a frequency of 1 Hz recorded the angle of the upper arm with respect to the line of gravity in six 15° intervals from 0° to 90°, and a seventh interval for angles above 90°. After exclusion of two subjects with less than four measured working days, the data set comprised measurements from 23 subjects. For subjects with five working days, four days were randomly selected in order to obtain a balanced data set for further processing. Some short periods of missing data within recorded working days occurred, in all 299 minutes out of a total of 126,824 minutes, and they were replaced using linear interpolation [14].

**Figure 1:**
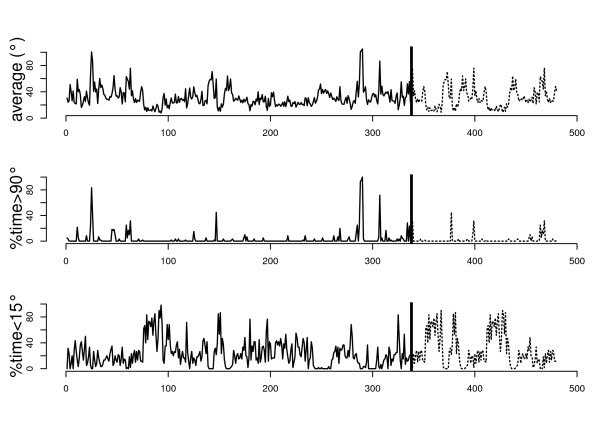
**Upper arm postures during an illustrative full shift; minute-by-minute values of average elevation, percentage time above 90°, and percentage time below 15° (top to bottom).** The vertical line at minute #337 separates original data (solid curve) from simulated data (dashed curve) added to achieve a full 480 minute shift.

The recordings from the different working days were of different duration, ranging from 240 minutes to 721. Most working days were close to 480 minutes. In a block bootstrap procedure, blocks of 30 minutes of simulated data were added to all days shorter than 480 minutes by resampling from the available data of the same day, until the day contained at least 480 minutes of data. All working days longer than 480 minutes were truncated at 480 minutes. This padding and truncating procedure has been described in detail and validated in a previous paper [14]. The described resulting data set was balanced and consisted of 480 minutes of upper arm elevation recordings from each of four days in each of 23 subjects. From this data set, we calculated minute-by-minute values of three exposure variables*: average elevationpercentage time above 90°* (percentage of time spent with an arm elevation larger than 90°), and *percentage time below 15°* (percentage of time spent with an arm elevation less than 15*°*)*.* Prior to assessing average angles, each Abduflex interval recording was replaced by the central angle of that interval, except for angles above 90° that were assigned the value 105°. Figure 1 shows an example of the three posture variables during a working day of a car mechanic.

### Statistical model

The three “parent” exposure data sets (one data set for each exposure variable) were analyzed using a standard hierarchical random effects model as follows:

(1) $$y_{\mathrm{ijk}}=\mu +\alpha_{i}+\beta_{j(i)}+\varepsilon_{k(ij)}\text{,}$$

i=1, 2,.., a j=1, 2, 3, 4 k=1,2,.., n

where *y*_*ijk*_ is the *k*:th observation of exposure on the *i*:th subject on that subject’s *j*:th working day*, μ* is the true mean exposure of the population, *α*_*i*_ is the random effect on exposure of the *i*:th subject, *β*_*j(i)*_ is the random effect on exposure of the *j*:th day within person *i*, and *ϵ*_*k(ij)*_ (the error term) is the random error for observation *k* within day *j* for person *i*. The variables *α*_*i*_*, β*_*j(i)*_ and *ϵ*_*k(ij)*_ are assumed to be independent and normally distributed with zero mean and variances *σ*^*2*^_*BS*_ (the between-subjects variance)_*,*_*σ*^*2*^_*BD*_ (the between-days variance) and *σ*^*2*^_*WD*_ (the within-day variance between 1-minute exposure values), respectively - for all *ij* and *k*. To examine the assumptions of the model, values of *α*_*i*_*, β*_*j(i)*_ and *ϵ*_*k(ij)*_ were predicted for each exposure variable [8]. Variance components and mean exposures were estimated using the restricted maximum likelihood method [8]. The autocorrelation function of lag 1, 2, 3, 4, 5, and 10 minutes was estimated for data within each working day and these values were then averaged across working days and subjects for each lag of the autocorrelation function. Autocorrelation describes the similarity of observations separated by a specific time span (the lag) in terms of a standard Pearson product–moment correlation coefficient. Hence, autocorrelation ranges between −1 and 1, where a value of 0 implies no correlation at all, and −1 and 1 show perfect predictability of data ahead in time.

### Sampling strategies

By simulation, we investigated a selection of sampling strategies defined by the number of sampled subjects (*n*_*s*_), the total sampling time collected from each subject (*t*_*tot*_, in minutes), the number of working days per subject (*n*_*d*_) among which the sampled time periods were distributed, and the size of sampled time blocks within each working day (*t*_*b*_, in minutes). Blocks were dispersed across the day at *random*, or using a *fixed time interval* schedule. Table 1 summarizes the investigated sampling strategies. For example, one sampling strategy used *n*_*s*_ = 10 subjects, *t*_*tot*_ = 120 minutes, *n*_*d*_ = 2 days, *t*_*b*_ = 15 minutes and *random sampling*. This implied sampling 120 minutes from each of 10 subjects, evenly allocated to two days. The 60 minutes from each day were sampled in four blocks, each 15 minutes long, and the blocks were randomly allocated within the day, with no overlap. In the corresponding fixed interval strategy, the first of the four blocks was randomly positioned within the first 105 minute of the working day, i.e. one fourth of the non-sampled time during that day. The remaining three blocks were distributed evenly across the day with a fixed time interval of 105 minutes between each block.

**Table 1:** Parameters characterizing the investigated sampling strategies

**Parameter**	**Values**
Number of subjects, *n*_*s*_	10,20
Sampling time per subject, *t*_*tot*_ (minutes)	60, 120, 240, 480
Number of days, *n*_*d*_	2, 4
Block size, *t*_*b*_ (minutes)	1, 15, 60, 240
Dispersion of blocks within days	random, fixed interval

All 128 (2x4x2x4x2) possible combinations of the parameter values in Table 1 were considered for each of the three posture variables. However, several of the combinations do not represent conceivable sampling strategies. For example, the block size cannot exceed the total sampling time per subject. In addition, when sampling one block per day, any fixed interval strategy is identical to the corresponding random strategy and therefore redundant. After rejecting impossible and redundant combinations, 80 sampling strategies remained. The performance of all 80 strategies was determined for each of the three posture variables using a bootstrap procedure.

### Bootstrapping

To investigate the performance of the 80 sampling strategies, non-parametric bootstrapping was performed on 3-level hierarchical data sets, levels being subjects, days and measurements within days [29]. Five thousand bootstrap runs were carried out for each combination of strategy and posture variable. In Liv et al. [14], 10000 bootstrap runs were used for investigating the precision of mean values, but estimating variance components is more computationally intensive, and therefore we settled for 5000 bootstrap runs that seemed sufficient to ensure stable estimates. In each bootstrap repeat, *n*_*s*_ subjects were selected with replacement from the parent data set. Within each selected subject, *n*_*d*_ days were then selected without replacement, and from within each selected working day *t*_*tot*_/*n*_*d*_ minutes were selected without replacement, using the block size prescribed by the sampling strategy. Thus, each bootstrap run resulted in a simulated data set, the variance components of which were estimated using the random effects model explained above (equation (1)). For each sampling strategy and variable, the *accuracy* of the estimators of the three variance components was expressed as bias. The bias was calculated by subtracting the mean of the estimates of that variance component across all 5000 bootstrap runs from the corresponding “true” variance component as estimated from the “parent” data set. As a measure of the *precision* of each variance component, a 90% prediction interval was estimated using the 5- and 95-percentiles of the empirical distribution of the 5000 bootstrap estimates of that variance component. A future estimate of a particular variance component will fall within the prediction interval with a probability of 90%. All simulation and estimation procedures were performed in the program R [30]; restricted maximum likelihood estimates of the variance components were obtained by the function lmer4.

## Results

For all three exposure variables, the total variability of the “parent” data set was dominated by the within-day variance component (Table 2; data also reported in Liv et al. 2011). The autocorrelation at lag 1 ranged between 0.51 and 0.55 for the three exposure variables, which demonstrates that the assumption of independence in the error term of the statistical model (equation 1) was violated.

**Table 2:** Estimates of mean exposure values, variance components and autocorrelation parameters in the parent data set

**Parameter**	**Average elevation**	**%time >90°**	**%time <15°**
***μ***	29.2°	4.7	32.5
***σ***^***2***^_***BS***_	22.2 (°)^2^	3.0	152.8
***σ***^***2***^_***BD***_	9.2 (°)^2^	4.0	65.6
***σ***^***2***^_***WD***_	234.9 (°)^2^	164.7	616.1
***ρ(1)***	0.55	0.52	0.51
***ρ(2)***	0.37	0.34	0.33
***ρ(3)***	0.29	0.26	0.26
***ρ(4)***	0.23	0.22	0.22
***ρ(5)***	0.19	0.17	0.18
***ρ(10)***	0.09	0.08	0.09

*μ*, mean value; *σ*^*2*^_*BS,*_*σ*^*2*^_*BD*_*, σ*^*2*^_*WD,*_ variances between subjects, between days and within days; *ρ(h)*, sample autocorrelation at lag *h*.

In Table 3, accuracy and precision of the estimated variance components for *percentage time above 90°* are presented for the subset of all investigated sampling strategies for which *n*_*s*_ = 10 or 20, *n*_*d*_ = 2 or 4, *t*_*tot*_ = 120 or 480 minutes and *t*_*b*_ = 1, 15 or 60 minutes. A table containing results from all investigated sampling strategies and exposure variables is provided as an Additional file 1: *Liv_complete_results.pdf*.

**Table 3:** **Bias [90% prediction intervals] of variance component estimates for*percentage time above 90°*for a subset of the investigated sampling strategies**

***n***_***s***_			**10**	**10**	**10**	**10**	**20**	**20**	**20**	**20**
***t***_***tot***_			**120**	**120**	**480**	**480**	**120**	**120**	**480**	**480**
***n***_***d***_			**2**	**4**	**2**	**4**	**2**	**4**	**2**	**4**
3a. Between-subjects variance, *σ*^*2*^_*BS*_										
***t***_***b***_	**1**	**r**	0.0	***−0.2***	−0.1	−0.1	−0.1	−0.1	−0.2	−0.2
			[−3.0, 5.3]	***[−3.0, 4.1]***	[−3.0, 4.1]	[−2.8, 3.0]	[−3.0, 3.6]	[−2.6, 2.7]	[−2.9, 2.7]	[−2.0, 1.9]
	**1**	**f**	0.0	−0.1	0.0	−0.3	−0.1	−0.2	0.0	−0.3
			[−3.0, 5.0]	[−3.0, 3.8]	[−3.0, 4.2]	[−2.9, 2.5]	[−3.0, 3.4]	[−2.4, 2.5]	[−2.7, 2.7]	[−2.1, 1.7]
	**15**	**r**	0.8	0.4	−0.1	−0.2	0.2	0.0	−0.2	−0.2
			[−3.0, 11.1]	[−3.0, 9.7]	[−3.0, 4.8]	[−3.0, 4.1]	[−3.0, 7.6]	[−3.0, 6.3]	[−3.0, 3.1]	[−2.6, 2.6]
	**15**	**f**	0.5	0.3	−0.5	−0.6	0.1	0.0	−0.6	−0.7
			[−3.0, 9.9]	[−3.0, 9.4]	[−3.0, 3.3]	[−3.0, 2.8]	[−3.0, 6.6]	[−3.0, 6.2]	[−3.0, 2.1]	[−2.8, 1.6]
	**60**	**r**	1.6		0.0	−0.1	0.7		−0.2	−0.2
			[−3.0, 17.3]		[−3.0, 5.8]	[−3.0, 5.5]	[−3.0, 11.5]		[−3.0, 3.7]	[−3.0, 3.6]
	**60**	**f**			−0.3	0.1			−0.4	−0.1
					[−3.0, 5.0]	[−3.0, 5.7]			[−3.0, 3.2]	[−3.0, 3.6]
3b. Between-days variance, *σ*^*2*^_*BD*_										
***t***_***b***_	**1**	**r**	−0.2	−0.1	−0.1	0.0	0.0	0.0	0.0	0.0
			[−4.0, 6.1]	[−4.0, 5.3]	[−3.4, 4.8]	[−2.5, 3.2]	[−3.5, 4.6]	[−3.3, 3.8]	[−2.6, 3.3]	[−1.9, 2.3]
	**1**	**f**	−0.8	−1.1	−0.3	−0.3	−0.7	−1.1	−0.2	−0.3
			[−4.0, 5.3]	[−4.0, 3.7]	[−3.4, 4.7]	[−2.8, 2.9]	[−4.0, 3.6]	[−4.0, 2.4]	[−2.8, 3.4]	[−2.2, 1.9]
	**15**	**r**	8.2	20.2	0.9	3.5	8.7	20.3	1.0	3.6
			[−1.8, 24.5]	[4.0, 44.6]	[−3.0, 6.7]	[−0.7, 9.0]	[0.5, 20.5]	[7.5, 37.5]	[−2.1, 5.1]	[0.5, 7.5]
	**15**	**f**	7.1	19.2	0.1	2.6	7.3	19.4	0.3	2.7
			[−1.9, 20.9]	[3.9, 41.9]	[−3.3, 5.0]	[−1.0, 7.4]	[0.1, 16.9]	[7.4, 35.1]	[−2.6, 4.0]	[−0.1, 6.2]
	**60**	**r**	17.2		2.0	7.3	17.6		2.1	7.4
			[−0.5, 51.0]		[−2.7, 9.0]	[0.7, 17.0]	[2.7, 42.0]		[−1.6, 7.2]	[2.4, 14]
	**60**	**f**			1.3	7.7			1.5	7.9
					[−2.8, 7.2]	[1.0, 17.9]			[−1.8, 6.0]	[2.6, 14.7]
3c. Within-day variance, *σ*^*2*^_*WD*_										
***t***_***b***_	**1**	**r**	0.1	−0.1	−0.9	0.2	0.5	0.2	−0.3	−0.1
			[−50.5, 54.8]	[−46.7, 50.8]	[−42.9, 42.3]	[−37.3, 38.9]	[−35.6, 39.1]	[−33.1, 34.9]	[−29.8, 31.4]	[−27.2, 27.1]
	**1**	**f**	0.5	−0.2	1.1	0.5	0.6	0.4	1.4	0.3
			[−48.9, 54.1]	[−44.3, 47.7]	[−42.0, 46.5]	[−35.9, 38.4]	[−34.3, 37.3]	[−32.2, 33.4]	[−29.9, 33.6]	[−26.2, 25.9]
	**15**	**r**	−8.8	−19.0	0.7	−2.1	−6.9	−18.7	0.6	−1.9
			[−72.9, 61.4]	[−74.3, 43.3]	[−43.7, 47.3]	[−44.3, 42.9]	[−53.8, 43.5]	[−60.0, 26.1]	[−31.6, 34.4]	[−31.5, 29.5]
	**15**	**f**	−8.5	−16.7	−2.4	−6.2	−9.2	−17.6	−1.5	−5.9
			[−70.0, 60.7]	[−73.8, 46.3]	[−44.4, 43.5]	[−44.6, 35.2]	[−55.3, 39.9]	[−59.3, 26.5]	[−31.0, 29.2]	[−33.8, 23.6]
	**60**	**r**	−13.0		1.2	−3.2	−13.3		1.0	−2.7
			[−80.6, 65.7]		[−45.6, 53.2]	[−47.5, 45.8]	[−61.0, 39.5]		[−34.0, 36.9]	[−34.9, 32.5]
	**60**	**f**			−1.1	3.1			−0.5	4.1
					[−47.2, 47.9]	[−42.1, 52.8]			[−32.0, 34.0]	[−29.0, 39.7]

a, b, c: between-subjects, between-days, and within-day variance.

*n*_*s*_, number of subjects; *t*_*tot*_, total sampling time per subject (minutes); *n*_*d*_, number of days per subject; *t*_*b*_, size of sampling blocks (minutes); r, random sampling; f, fixed interval sampling.

Bias and prediction intervals are presented relative to the “true” variance components of the parent data set, i.e. *σ*^*2*^_*BS*_ = 3.0, *σ*^*2*^_*BD*_ = 4.0 and *σ*^*2*^_*WD*_ = 164.7 (cf. Table 2). Thus, the result in ***bold and italics*** tells that for the sampling strategy (*n*_*s*_, *t*_*tot*_, *n*_*d*_, *t*_*b*_) = (10, 120, 4, 1) with random distribution of blocks, *σ*^*2*^_*BS*_ was downward biased by 0.2, i.e. its average estimated value was 2.8, and the 90% prediction interval, presented as [−3.0, 4.1], reached from 3.0-3.0 to 3.0 + 4.1, i.e. from 0.0 to 7.1.

**Figure 2:**
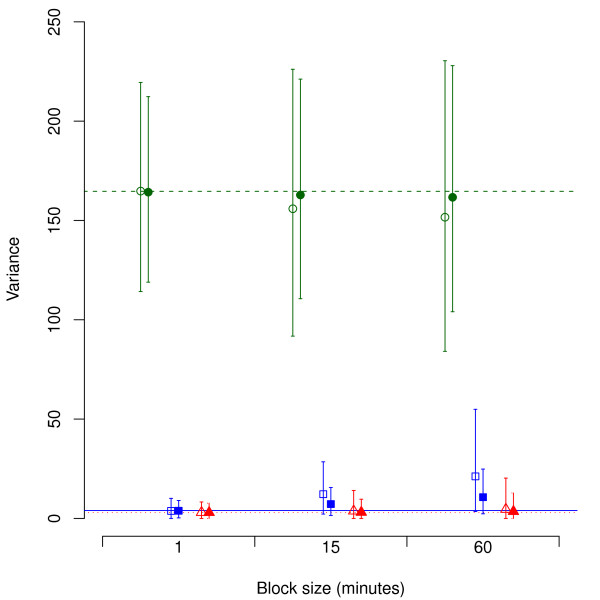
**Estimated variance component mean values (error bars: 90% prediction intervals) resulting from simulations of six different sampling strategies assessing percentage time above 90°.** All six strategies used random sampling from *n*_*s*_ = 10 subjects, approached for *n*_*d*_ = 2 days each. Green circles, blue squares and red triangles show within-day, between-days and between-subjects variance, respectively. Unfilled and filled symbols show strategies with *t*_*tot*_ = 120 minutes and *t*_*tot*_ = 240 minutes, respectively. Red dashed, blue solid and green dotted lines represent the “true” within-day, between-days and between-subjects variance, respectively, according to the parent data set

For strategies with a block size of 1 minute, bias was absent or very small. In general, when block size increased, a stronger negative bias appeared for the within-day variance, and a stronger positive bias for the between-days variance (Table 3; illustrated in Figure 2). This effect was more pronounced with a smaller total sample size per subject (Figure 2). The accuracy of the between-subjects variance was not affected by the sampling strategy to the same extent as the variance between and within days.

As expected, precision increased (i.e. the 90% prediction interval of the variance components narrowed) when more data was sampled (Table 3). At a particular total sampling time, *t*_*tot*_, the prediction interval widened, i.e. the variance components were less precise, for larger block sizes. This effect appears clearly in Figure 2. In general, distributing a particular sampling time across four days (*n*_*d*_ = 4) led to better precision for all three variance components than when distributing the same sampling time across two days (*n*_*d*_ = 2). However, distributing sampling time across more days also resulted in less accurate variance components when the block size was large. We did not find obvious systematic differences between variance components obtained by random and fixed-interval sampling. Very similar patterns were found for the other two exposure variables.

## Discussion

In the present study, different sampling strategies were evaluated with respect to accuracy and precision of variance components for three posture variables for car mechanics. The results showed the consequences of violating theoretical assumptions behind the random effects model; inaccurate results were caused by individual samples being time-dependent within working days (autocorrelation). The present study used a bootstrap method for investigating the performance of sampling strategies that is also applicable in other occupational settings and for other exposure variables.

The present study showed that sampling data in large time blocks may lead to inaccuracy and imprecision in estimates of variance components. This was particularly prominent for strategies where small fractions of working days were sampled. Variance component estimates were particularly biased for strategies with small sample sizes *and* large block sizes. For *percentage time above 90°*, negative biases of up to 26% of the size of the “true” within-day variance component and positive biases of up to 1110% of the size of the “true” between-days variance component were observed. However, in many cases the total error in the variance component estimates was dominated by imprecision rather than inaccuracy. For the within-day variance component, which was estimated at 164.7 in the original data set, the 90% prediction interval ranged from 137 to 191 for the sampling strategy giving the best precision (*n*_*s*_ = 20, *t*_*tot*_ = 480, *n*_*d*_ = 4, *t*_*b*_ = 1). For the within-day variance component, the median width of the 90% prediction interval across all investigated sampling strategies was 89 while the median bias of the variance component was 1.4. Thus, our results suggested that imprecision will often be a more serious problem than inaccuracy for studies of the sizes simulated here. The results further indicated that the sample sizes investigated by us might not be sufficient to retrieve variance components with a satisfying precision. In occupational epidemiology, variance components are required for designing efficient exposure measurement strategies, and when combined with information on costs associated with data collection, they give a basis for deciding on efficient budget allocation [31-34]. Variance components can guide the selection of targets for interventions to reduce suspected hazardous exposures [2]; they are used in assessments of clinical reliability [35], and they are necessary inputs in conventional power analysis of, for instance, studies addressing exposure differences between groups or effects of an intervention [4,6]. The present paper clearly illustrates that the results of these applications of estimated variance components can be very uncertain, in particular if the estimates have been based on short and continuous exposure samples. This caveat is rarely addressed in the literature. Estimated variances are known to follow a positively skewed distribution; this was apparent even in the present study. Hence, a sample estimate of a variance is more likely too small compared to the true value than too large. Using an estimated variance in a power analysis of a planned intervention study will therefore more often lead to too “optimistic” (small) predictions of the necessary study size than to too “pessimistic”. The error may be considerable, as illustrated by the wide prediction intervals on variances in the present study. In order to account for variance estimation uncertainty in power analyses, some authors have suggested to use the 80^th^ percentile of the expected distribution of variance estimates as an input rather than the actual variance estimate [36]. A particular challenge appears if variance components *per se* are the exposure variables of interest, for instance in studies of exposure variation [6,7]. Variances are not normally distributed, and a conventional power analysis, which requires data to have this property, is not applicable. Developing power analysis procedures for studies addressing variance components is an interesting issue for further research.

The epidemiologic study, for which the data was originally sampled, attempted a random collection of subjects and working weeks [21,26,27]. The present data is therefore a likely representative sample of car mechanics’ exposure to elevated upper arms. The original study also included measurements on house painters and machinists [21,26,27]. The car mechanics, spending on average 4.7% time with the right arm elevated above 90°, worked more with elevated arms than machinists (1.6% time >90°), but less than house painters (8.8% time >90°). The size of exposure variability in the three groups differed in a similar fashion; the car mechanics showed more variability than the machinists did, but less than the house painters did. The autocorrelation function for *percentage time above 90°* at lag 1 was 0.31 for machinists and 0.46 for house painters, compared to an autocorrelation of 0.52 for the car mechanics [14]. This implies violation of the assumption of independence in the error term also for machinists and house painters. This leads us to believe that the principal effects of sampling strategy on variance component estimators shown in the present study are relevant also to data collections of other exposure variables and in other occupational groups. The magnitude of these effects, however, probably varies between variables and occupational groups, and our numerical results should therefore be applied outside the group of car mechanics only with great caution.

Variability of upper arm elevation has been reported in the literature for other occupations [6,22,37], but the posture variables were different from the ones used in the present study and accuracy and precision of the reported variance components were not explored. Consistent with our findings, estimates of variance components were shown to be associated with considerable imprecision in previous studies on muscle activity during assembly work [3,4] and on posture and electromyography data from short-cycle manual handling [6].

The present study determined exposure variability between and within subjects using a random effects model, which assumes that effects are uncorrelated. Results showed that this assumption was violated since the car mechanics exhibited considerable autocorrelation between measurements within a working day. As demonstrated by David [38], the ordinary sample variance estimator, $\sum_{i}{\left(x_{i}-\bar{x}\right)}^{2}/(n-1)$, underestimates the population variance if observations are not independent and if the sample size is not large. An equivalent effect can be expected on the estimator for within-day variance. This is a likely explanation why within-day variance estimates were inaccurate for sampling strategies with larger block sizes, while they were not for strategies with block size 1, where observations will be (close to) unaffected by autocorrelation. Since variance components are partitions of the total (constant) variance present in the data, a negative bias in the within-day variance estimate propagates to the other variance components, in particular showing up as a positive bias in the between-days variance. When block size increases, the time span between the observations in the sample decreases. Hence, the sample will be more autocorrelated, which leads to a larger bias. We believe that increased autocorrelation explains the occasional larger bias of variance components estimated by strategies where a particular sampling time was distributed across four days rather than two. This will lead to a smaller sampling time per day and – if the block size is large – to a more dominant effect of autocorrelation.

Violations of other assumptions of the random effects model (equation 1) than independence also occurred. Visual inspections of plots of predicted values of the random effects and their residuals suggested that the assumption of constant variance across subjects and days was violated in some cases. The residuals also had positively skewed distributions. Although the model in equation (1) makes no assumptions of the distributional form of the random effects, the REML estimators that we used to estimate the variance components assume normal distributions. However, REML estimators are identical to ANOVA estimators when data is balanced [39], and as ANOVA estimators are not based on distributional assumptions we do not consider this to be a problem. We did not transform the exposure data because we could not identify a transformation that improved the fit of the random effects model to any noticeable extent.

More complex random effects models are available that can model time dependence in the error term by incorporating correlation structures according to different time series models, like autoregressive models (AR) or moving average models (MA) [40]. A successful fit of such a model might result in unbiased measures of variability between and within subjects. However, for data sets as large as that used in the present study (23 subjects*4 days*480 minutes = 44160 observations), computations with very large variance and covariance matrices will be involved. Thus, it may not be possible to fit these models. Moreover, identifying a reasonable model of the time dependence of our posture variables is not a trivial matter.

A parametric model for the structure of the arm elevation data was not available so parametric bootstrapping [41] was not an option. While procedures for non-parametric bootstrapping for hierarchical data have mainly been discussed in the context of two-level data sets [29,42-44], a recent paper by Ren et al. [45] addressed non-parametric bootstrapping for data sets with three levels or more. The paper concluded that units at the first level (here subjects) should be selected with replacement while units at the two lower levels (here days and quanta within days) should be selected without replacement; this was the procedure used in the present paper.

## Conclusions

If exposure data are autocorrelated within days, which is probably a common case for biomechanical exposures in occupational settings, limited sampling may lead to inaccuracy in estimated variance components, in addition to a large imprecision. Applying a larger total sample size can improve both accuracy and precision, and further improvements can be obtained by distributing samples well across time.

Since inaccuracy and imprecision of variance component estimators is an issue in limited exposure sampling, occupational research and practice addressing variance components for descriptive, epidemiologic or intervention purposes may face the need for studies of a considerable size – larger than commonly done – if this inaccuracy and imprecision is to be reduced to levels normally pursued in studies addressing mean exposures.

Our findings lead us to the following guidance for sampling strategies addressing variance components:

· A larger data sample will be required to reach a satisfying precision of variance components than when the purpose is to estimate a mean value with good precision.

· Distributing a within-day sample across the whole day and in small blocks will lead to variance component estimators with a better accuracy and precision than if the sample is collected in larger time blocks.

· Increasing the total within-day sample size increases the accuracy and precision of variance component estimators and reduces the adverse effects of sampling in larger blocks.

· Distributing a certain total sample size across more days may result in more precise but in some cases also less accurate variance component estimators than if the sample is distributed across fewer days.

## Competing interests

The authors declare that they have no competing interests.

## Authors’ contributions

PL designed most of the analytical procedures, performed the sampling simulations and drafted major parts of the manuscript. SEM conceived the study, contributed to data analysis, and drafted significant parts of the manuscript. SWS was responsible for the data collection, contributed to interpretation of data and drafted significant parts of the manuscript. All authors read and approved the final manuscript.

## Pre-publication history

The pre-publication history for this paper can be accessed here:

http://www.biomedcentral.com/1471-2288/12/58/prepub

## Supplementary Material

Additional file 1 **Tables A1-A6. Bias [90% prediction intervals] of variance component estimates for all three posture variables and all investigated sampling strategies.** Table A1. Average elevation, *n*_*s*_ = 10. A1a, A1b, A1c: between-subjects, between-days, and within-day variance. Table A2. Average elevation, *n*_*s*_ = 20. A2a, A2b, A2c: between-subjects, between-days, and within-day variance. Table A3. Percentage time above 90°, *n*_*s*_ = 10. A3a, A3b, A3c: between-subjects, between-days, and within-day variance. Table A4. Percentage time above 90°, *n*_*s*_ = 20. A4a, A4b, A4c: between-subjects, between-days, and within-day variance. Table A5. Percentage time below 15°, *n*_*s*_ = 10. A5a, A5b, A5c: between-subjects, between-days, and within-day variance. Table A6. Percentage time below 15°, *n*_*s*_ = 20. A6a, A6b, A6c: between-subjects, between-days, and within-day variance. *n*_*s*_, number of subjects; *t*_*tot*_, total sampling time per subject (minutes); *n*_*d*_, number of days per subject; *t*_*b*_, size of sampling blocks (minutes); r, random sampling; f, fixed interval sampling. The prediction intervals are presented relative to the “true” variance components of the parent data set (cf. Table 3). Liv_complete_results.pdf. Click here for file
