# Supplementary material for: Accuracy and precision of variance components in occupational posture recordings: a simulation study of different data collection strategies
Source: BMC Med Res Methodol. 2012 Jun 18;12:58. doi: 10.1186/1471-2288-12-58 (PMC3377541; doi:10.1186/1471-2288-12-58)
Supplement: Additional file 1 — Tables A1-A6. Bias [90% prediction intervals] of variance component estimates for all three posture variables and all investigated sampling strategies. Table A1. Average elevation, ns = 10. A1a, A1b, A1c: between-subjects, between-days, and within-day variance. Table A2. Average elevation, ns = 20. A2a, A2b, A2c: between-subjects, between-days, and within-day variance. Table A3. Percentage time above 90°, ns = 10. A3a, A3b, A3c: between-subjects, between-days, and within-day variance. Table A4. Percentage time above 90°, ns = 20. A4a, A4b, A4c: between-subjects, between-days, and within-day variance. Table A5. Percentage time below 15°, ns = 10. A5a, A5b, A5c: between-subjects, between-days, and within-day variance. Table A6. Percentage time below 15°, ns = 20. A6a, A6b, A6c: between-subjects, between-days, and within-day variance. ns, number of subjects; ttot, total sampling time per subject (minutes); nd, number of days per subject; tb, size of sampling blocks (minutes); r, random sampling; f, fixed interval sampling. The prediction intervals are presented relative to the “true” variance components of the parent data set (cf. Table 3). Liv_complete_results.pdf. [file 1471-2288-12-58-S1.pdf]

A1a. Average elevation, n_s = 10. Between-subjects variance, σ^2_{BS}

		t_{tot}	**60**	**60**	**120**	**120**	**240**	**240**	**480**	**480**
		n_d	**2**	**4**	**2**	**4**	**2**	**4**	**2**	**4**
t_b	**1**	**r**	-1.0<br>[-22.1, 29.4]	-1.3<br>[-19.7, 25.4]	-1.2<br>[-20.7, 27.3]	-1.4<br>[-18.2, 23.4]	-1.0<br>[-19.7, 27.7]	-1.0<br>[-17.5, 24.1]	-0.5<br>[-19.1, 28.3]	-1.1<br>[-17.0, 22.5]
	**1**	**f**	-0.7<br>[-21.2, 29.8]	-1.4<br>[-19.1, 24.4]	-0.7<br>[-19.4, 28.2]	-0.7<br>[-17.7, 24.7]	-1.1<br>[-19.6, 27.7]	-0.6<br>[-16.9, 24.2]	-1.0<br>[-19.2, 27.7]	-1.4<br>[-17.1, 22.3]
	**15**	**r**	-0.6<br>[-22.3, 39.7]	-0.4<br>[-22.3, 39.7]	-0.6<br>[-22.3, 34.4]	-0.9<br>[-22.3, 29.9]	-1.1<br>[-22.3, 29.8]	-1.1<br>[-19.7, 26.8]	-1.3<br>[-20.5, 27.5]	-1.1<br>[-18.2, 24.8]
	**15**	**f**	0.2<br>[-22.3, 40.7]		-0.7<br>[-22.3, 32.5]	-1.2<br>[-22.3, 29.7]	-0.8<br>[-22.3, 29.2]	-0.7<br>[-19.0, 26.3]	-0.7<br>[-19.5, 28.9]	-0.7<br>[-18.0, 24.4]
	**60**	**r**			-0.9<br>[-22.3, 37.4]		-1.4<br>[-22.3, 32.4]	-1.7<br>[-22.3, 28.0]	-1.3<br>[-21.8, 29.6]	-1.5<br>[-19.6, 25.2]
	**60**	**f**					-2.4<br>[-22.3, 29.1]		-1.8<br>[-22.2, 28.5]	-2.3<br>[-19.3, 22.9]
	**240**	**r**							-1.6<br>[-22.3, 30.0]	
	**240**	**f**								

A1b. Average elevation, n_s = 10. Between-days variance, σ^2_{BD}

		t_{tot}	60	60	120	120	240	240	480	480
		n_d	2	4	2	4	2	4	2	4
t_b	1	r	0.1 [-9.2, 14.3]	0.0 [-9.2, 12.1]	-0.1 [-8.3, 11.1]	0.1 [-6.8, 8.1]	0.0 [-6.9, 9.6]	0.0 [-5.3, 6.4]	0.0 [-6.5, 8.7]	0.0 [-4.6, 5.4]
	1	f	-1.8 [-9.2, 10.4]	-1.9 [-9.2, 9.5]	-1.6 [-8.9, 8.3]	-2.0 [-8.1, 5.2]	-0.7 [-7.4, 8.5]	-1.4 [-6.3, 4.1]	-0.2 [-6.6, 8.6]	-0.6 [-5.1, 4.6]
	15	r	27.1 [0.9, 64.4]	60.0 [23.1, 111.0]	12.1 [-3.2, 34.1]	28.2 [9.6, 52.7]	4.9 [-4.9, 18.7]	12.6 [2.5, 25.4]	1.5 [-5.8, 11.8]	4.9 [-1.4, 12.4]
	15	f	24.7 [-0.5, 62.8]		10.6 [-3.6, 30.2]	25.8 [8.2, 47.9]	3.5 [-5.5, 16.1]	11.0 [1.2, 22.5]	1.1 [-6.0, 10.1]	3.4 [-2.2, 10.2]
	60	r			23.6 [0.8, 58.1]		9.3 [-3.5, 27.1]	24.2 [7.5, 45.6]	2.8 [-5.3, 14.0]	9.7 [0.8, 20.9]
	60	f					9.0 [-3.7, 26.7]		3.5 [-5.3, 16.3]	9.0 [0.5, 19.6]
	240	r							4.0 [-5.1, 16.5]	
	240	f								

A1c. Average elevation, n_s = 10. Within-day variance, σ^2_{WD}

		t_{tot}	60	60	120	120	240	240	480	480
		n_d	2	4	2	4	2	4	2	4
t_b	1	r	-0.4 [-47.8, 48.9]	-0.4 [-43.6, 45.7]	0.1 [-37.5, 41.8]	0.1 [-34.9, 36.4]	-0.4 [-35.6, 35.7]	0.4 [-30.7, 32.7]	-0.7 [-33.3, 31.6]	0.1 [-28.2, 28.3]
	1	f	1.9 [-43.8, 49.2]	2.2 [-39.4, 46.6]	1.6 [-35.6, 40.7]	1.1 [-32.6, 36.1]	1.2 [-32.8, 36.0]	1.6 [-27.2, 30.9]	1.8 [-31.3, 35.0]	1.5 [-25.7, 29.1]
	15	r	-26.2 [-84.8, 42.0]	-59.5 [-106.6, -7.3]	-11.8 [-62.2, 42.2]	-26.6 [-70.7, 21.3]	-3.4 [-44.6, 40.2]	-11.2 [-49.5, 28.6]	0.0 [-33.8, 35.2]	-3.6 [-35.2, 29.9]
	15	f	-23.2 [-82.9, 43.9]		-10.3 [-57.7, 40.9]	-23.2 [-67.3, 23.5]	-3.9 [-42.7, 38.4]	-10.9 [-46.6, 28.4]	-0.6 [-35.0, 34.9]	-5.0 [-35.1, 26.8]
	60	r			-19.6 [-74.1, 40.0]		-5.5 [-49.7, 44.5]	-19.6 [-59.4, 23.1]	0.1 [-36.6, 38.2]	-5.7 [-39.3, 30.8]
	60	f					1.4 [-42.4, 49.4]		-1.8 [-36.3, 32.4]	0.6 [-32.6, 37.1]
	240	r							1.3 [-37.5, 41.4]	
	240	f								

A2a. Average elevation, n_s = 20. Between-subjects variance, σ^2_{BS}

		t_{tot}	**60**	**60**	**120**	**120**	**240**	**240**	**480**	**480**
		n_d	**2**	**4**	**2**	**4**	**2**	**4**	**2**	**4**
t_b	**1**	**r**	-0.9 [-17.2, 19.7]	-1.1 [-15.2, 16.4]	-1.3 [-16.2, 18.4]	-1.0 [-14.4, 16.3]	-1.2 [-15.5, 17.9]	-1.2 [-14.1, 15.6]	-1.2 [-15.3, 17.9]	-0.9 [-13.8, 15.4]
	**1**	**f**	-1.0 [-16.1, 19.2]	-1.4 [-15.3, 16.5]	-0.7 [-15.5, 18.8]	-1.1 [-14.2, 15.8]	-1.2 [-15.4, 18.0]	-0.9 [-13.4, 15.7]	-0.8 [-14.5, 17.2]	-0.9 [-13.5, 15.2]
	**15**	**r**	-0.8 [-22.3, 27.9]	-1.5 [-22.3, 23.9]	-1.3 [-20.1, 21.5]	-1.5 [-18.5, 19.9]	-1.4 [-17.0, 18.9]	-1.0 [-15.5, 17.4]	-1.1 [-15.6, 18.5]	-1.1 [-14.4, 16.2]
	**15**	**f**	-1.0 [-22.3, 27.0]		-0.9 [-19.2, 21.8]	-0.9 [-17.3, 19.2]	-1.2 [-16.5, 18.2]	-1.0 [-15.4, 18.0]	-0.8 [-15.1, 18.5]	-0.9 [-14.0, 16.7]
	**60**	**r**			-1.9 [-22.3, 24.7]		-1.6 [-20.6, 20.6]	-1.8 [-18.2, 19.1]	-1.4 [-16.4, 18.9]	-1.7 [-15.5, 15.9]
	**60**	**f**					-2.8 [-20.9, 17.8]		-1.7 [-16.3, 18.3]	-2.5 [-15.7, 14.1]
	**240**	**r**							-2.0 [-17.1, 17.4]	
	**240**	**f**								

A2b. Average elevation, n_s = 20. Between-days variance, σ^2_{BD}

		t_{tot}	60	60	120	120	240	240	480	480
		n_d	2	4	2	4	2	4	2	4
t_b	1	r	0.1 [-7.7, 10.2]	-0.1 [-7.6, 7.9]	0.1 [-6.2, 8.1]	0.0 [-4.9, 5.7]	0.0 [-5.3, 6.6]	0.0 [-3.9, 4.5]	0.0 [-4.9, 5.9]	0.0 [-3.4, 3.8]
	1	f	-2.0 [-9.2, 6.7]	-2.1 [-9.2, 5.4]	-1.5 [-7.1, 5.3]	-2.0 [-6.4, 2.7]	-0.6 [-5.8, 5.5]	-1.5 [-5.0, 2.4]	-0.3 [-5.1, 5.5]	-0.7 [-3.9, 2.8]
	15	r	28.1 [6.5, 56.6]	61.0 [31.9, 97.0]	12.6 [0.7, 27.8]	28.1 [14.1, 45.2]	5.2 [-2.6, 14.8]	12.6 [4.9, 21.5]	1.5 [-4.0, 8.4]	5.1 [0.4, 10.5]
	15	f	25.6 [5.1, 52.8]		11.0 [-0.1, 24.7]	25.9 [12.8, 41.0]	3.4 [-3.3, 11.9]	11.0 [3.9, 18.8]	1.2 [-4.1, 7.7]	3.5 [-0.6, 8.2]
	60	r			24.1 [5.9, 49.2]		9.8 [-0.4, 23.0]	24.1 [11.6, 38.8]	2.9 [-3.4, 11.0]	9.6 [3.1, 17.1]
	60	f					9.2 [-0.7, 22.4]		3.6 [-3.3, 12.4]	9.0 [2.9, 16.4]
	240	r								
	240	f								

A2c. Average elevation, n_s = 20. Within-day variance, σ^2_{WD}

		t_{tot}	60	60	120	120	240	240	480	480
		n_d	2	4	2	4	2	4	2	4
t_b	1	r	-0.1 [-32.5, 34.6]	0.1 [-30.7, 32.4]	0.3 [-27.3, 29.5]	0.1 [-25.3, 25.6]	0.1 [-25.1, 25.6]	0.1 [-21.9, 21.9]	-0.3 [-22.7, 23.1]	-0.2 [-20.4, 20.0]
	1	f	0.6 [-30.6, 32.9]	1.8 [-28.3, 33.3]	1.5 [-24.9, 28.1]	1.6 [-23.4, 26.0]	1.7 [-22.5, 26.3]	1.8 [-19.3, 23.0]	1.8 [-21.8, 24.9]	1.1 [-18.5, 20.3]
	15	r	-26.7 [-70.3, 20.2]	-59.4 [-94.1, -2.3]	-10.6 [-45.5, 27.5]	-26.3 [-58.8, 8.9]	-3.6 [-32.8, 26.8]	-10.8 [-37.7, 18.0]	-0.1 [-24.8, 25.1]	-3.4 [-26.3, 19.9]
	15	f	-22.9 [-65.8, 23.1]		-10.6 [-45.9, 26.4]	-24.0 [-56.5, 10.1]	-4.9 [-33.4, 24.8]	-11.3 [-36.2, 16.0]	-0.4 [-24.1, 23.5]	-4.7 [-26.1, 17.6]
	60	r			-19.4 [-56.9, 20.5]		-5.6 [-38.3, 28.3]	-19.7 [-48.9, 10.7]	0.2 [-26.7, 26.8]	-5.3 [-30.1, 21.0]
	60	f					0.9 [-31.5, 35.6]		-1.4 [-25.1, 23.1]	1.5 [-23.1, 26.8]
	240	r							0.9 [-26.3, 28.8]	
	240	f								

A3a. Percentage time above 90°, n_s = 10. Between-subjects variance, σ^2_{BS}

		t_{tot}	60	60	120	120	240	240	480	480
		n_d	2	4	2	4	2	4	2	4
t_b	1	r	0.2 [-3.0, 7.0]	0.0 [-3.0, 5.4]	0.0 [-3.0, 5.3]	-0.2 [-3.0, 4.1]	0.0 [-3.0, 4.7]	-0.2 [-3.0, 3.4]	-0.1 [-3.0, 4.1]	-0.1 [-2.8, 3.0]
	1	f	0.1 [-3.0, 6.0]	-0.2 [-3.0, 4.9]	0.0 [-3.0, 5.0]	-0.1 [-3.0, 3.8]	-0.2 [-3.0, 4.0]	-0.1 [-2.9, 3.2]	0.0 [-3.0, 4.2]	-0.3 [-2.9, 2.5]
	15	r	2.0 [-3.0, 19.4]	1.3 [-3.0, 16.4]	0.8 [-3.0, 11.1]	0.4 [-3.0, 9.7]	0.1 [-3.0, 6.7]	0.0 [-3.0, 6.2]	-0.1 [-3.0, 4.8]	-0.2 [-3.0, 4.1]
	15	f	2.0 [-3.0, 19.6]		0.5 [-3.0, 9.9]	0.3 [-3.0, 9.4]	-0.3 [-3.0, 5.0]	-0.3 [-3.0, 5.3]	-0.5 [-3.0, 3.3]	-0.6 [-3.0, 2.8]
	60	r			1.6 [-3.0, 17.3]		0.5 [-3.0, 9.8]	0.2 [-3.0, 8.6]	0.0 [-3.0, 5.8]	-0.1 [-3.0, 5.5]
	60	f					0.5 [-3.0, 10.3]		-0.3 [-3.0, 5.0]	0.1 [-3.0, 5.7]
	240	r							-0.1 [-3.0, 5.4]	
	240	f								

A3b. Percentage time above 90°, n_s = 10. Between-days variance, σ^2_{BD}

		t_{tot}	60	60	120	120	240	240	480	480
		n_d	2	4	2	4	2	4	2	4
t_b	1	r	-0.2 [-4.0, 7.8]	-0.3 [-4.0, 8.2]	-0.2 [-4.0, 6.1]	-0.1 [-4.0, 5.3]	-0.2 [-3.7, 5.0]	0.0 [-3.0, 3.9]	-0.1 [-3.4, 4.8]	0.0 [-2.5, 3.2]
	1	f	-1.1 [-4.0, 6.3]	-1.2 [-4.0, 7.4]	-0.8 [-4.0, 5.3]	-1.1 [-4.0, 3.7]	-0.5 [-3.9, 4.7]	-0.7 [-3.7, 3.3]	-0.3 [-3.4, 4.7]	-0.3 [-2.8, 2.9]
	15	r	18.3 [-1.2, 52.2]	42.7 [6.3, 101.0]	8.2 [-1.8, 24.5]	20.2 [4.0, 44.6]	3.2 [-2.5, 11.6]	9.0 [1.3, 19.9]	0.9 [-3.0, 6.7]	3.5 [-0.7, 9.0]
	15	f	17.6 [-1.1, 49.7]		7.1 [-1.9, 20.9]	19.2 [3.9, 41.9]	2.5 [-2.7, 10.8]	7.7 [0.9, 16.5]	0.1 [-3.3, 5.0]	2.6 [-1.0, 7.4]
	60	r			17.2 [-0.5, 51.0]		6.7 [-1.7, 20.8]	18.0 [3.6, 41.1]	2.0 [-2.7, 9.0]	7.3 [0.7, 17.0]
	60	f					7.3 [-1.5, 22.6]		1.3 [-2.8, 7.2]	7.7 [1.0, 17.9]
	240	r							2.5 [-2.4, 10.1]	
	240	f								

A3c. Percentage time above 90°, n_s = 10. Within-day variance, σ^2_{WD}

		t_{tot}	60	60	120	120	240	240	480	480
		n_d	2	4	2	4	2	4	2	4
t_b	1	r	-0.3 [-60.3, 64.4]	-0.7 [-55.8, 61.1]	0.1 [-50.5, 54.8]	-0.1 [-46.7, 50.8]	-0.4 [-45.7, 47.7]	0.5 [-40.4, 44.1]	-0.9 [-42.9, 42.3]	0.2 [-37.3, 38.9]
	1	f	0.7 [-57.1, 64.6]	1.0 [-53.4, 60.5]	0.5 [-48.9, 54.1]	-0.2 [-44.3, 47.7]	0.1 [-43.8, 46.8]	0.8 [-39.1, 42.9]	1.1 [-42.0, 46.5]	0.5 [-35.9, 38.4]
	15	r	-18.5 [-91.5, 70.6]	-43.5 [-100.5,22.5]	-8.8 [-72.9, 61.4]	-19.0 [-74.3, 43.3]	-1.8 [-54.1, 56.5]	-7.6 [-55.7, 46.3]	0.7 [-43.7, 47.3]	-2.1 [-44.3, 42.9]
	15	f	-16.5 [-92.3, 71.6]		-8.5 [-70.0, 60.7]	-16.7 [-73.8, 46.3]	-4.9 [-53.9, 50.2]	-9.2 [-55.7, 43.1]	-2.4 [-44.4, 43.5]	-6.2 [-44.6, 35.2]
	60	r			-13.0 [-80.6, 65.7]		-3.0 [-60.6, 63.3]	-13.3 [-63.5, 43.4]	1.2 [-45.6, 53.2]	-3.2 [-47.5, 45.8]
	60	f					4.4 [-54.4, 69.7]		-1.1 [-47.2, 47.9]	3.1 [-42.1, 52.8]
	240	r							3.1 [-46.6, 56.4]	
	240	f								

A4a. Percentage time above 90°, n_s = 20. Between-subjects variance, σ^2_{BS}

		t_{tot}	60	60	120	120	240	240	480	480
		n_d	2	4	2	4	2	4	2	4
t_b	1	r	0.0 [-3.0, 4.7]	-0.1 [-3.0, 3.7]	-0.1 [-3.0, 3.6]	-0.1 [-2.6, 2.7]	-0.1 [-3.0, 3.1]	-0.2 [-2.2, 2.2]	-0.2 [-2.9, 2.7]	-0.2 [-2.0, 1.9]
	1	f	-0.1 [-3.0, 4.0]	-0.2 [-3.0, 3.3]	-0.1 [-3.0, 3.4]	-0.2 [-2.4, 2.5]	-0.2 [-2.9, 2.7]	-0.1 [-2.1, 2.1]	0.0 [-2.7, 2.7]	-0.3 [-2.1, 1.7]
	15	r	1.2 [-3.0, 14.5]	0.6 [-3.0, 11.5]	0.2 [-3.0, 7.6]	0.0 [-3.0, 6.3]	-0.2 [-3.0, 4.5]	-0.2 [-3.0, 4.0]	-0.2 [-3.0, 3.1]	-0.2 [-2.6, 2.6]
	15	f	1.2 [-3.0, 13.7]		0.1 [-3.0, 6.6]	0.0 [-3.0, 6.2]	-0.6 [-3.0, 3.2]	-0.5 [-3.0, 3.1]	-0.6 [-3.0, 2.1]	-0.7 [-2.8, 1.6]
	60	r			0.7 [-3.0, 11.5]		0.0 [-3.0, 6.8]	-0.2 [-3.0, 6.1]	-0.2 [-3.0, 3.7]	-0.2 [-3.0, 3.6]
	60	f					0.1 [-3.0, 6.6]		-0.4 [-3.0, 3.2]	-0.1 [-3.0, 3.6]
	240	r							-0.3 [-3.0, 3.6]	
	240	f								

A4b. Percentage time above 90°, n_s = 20. Between-days variance, σ^2_{BD}

		t_{tot}	60	60	120	120	240	240	480	480
		n_d	2	4	2	4	2	4	2	4
t_b	1	r	-0.1<br>[-4.0, 5.6]	-0.4<br>[-4.0, 5.7]	0.0<br>[-3.5, 4.6]	0.0<br>[-3.3, 3.8]	0.0<br>[-2.9, 3.6]	-0.1<br>[-2.3, 2.7]	0.0<br>[-2.6, 3.3]	0.0<br>[-1.9, 2.3]
	1	f	-1.1<br>[-4.0, 4.4]	-1.5<br>[-4.0, 4.4]	-0.7<br>[-4.0, 3.6]	-1.1<br>[-4.0, 2.4]	-0.4<br>[-3.2, 3.2]	-0.7<br>[-3.0, 2.0]	-0.2<br>[-2.8, 3.4]	-0.3<br>[-2.2, 1.9]
	15	r	19.3<br>[2.6, 43.8]	44.2<br>[13.8, 86.1]	8.7<br>[0.5, 20.5]	20.3<br>[7.5, 37.5]	3.6<br>[-1.3, 10.5]	9.1<br>[3.2, 16.7]	1.0<br>[-2.1, 5.1]	3.6<br>[0.5, 7.5]
	15	f	18.6<br>[2.8, 42.8]		7.3<br>[0.1, 16.9]	19.4<br>[7.4, 35.1]	2.6<br>[-1.5, 8.3]	7.8<br>[2.8, 13.9]	0.3<br>[-2.6, 4.0]	2.7<br>[-0.1, 6.2]
	60	r			17.6<br>[2.7, 42.0]		7.3<br>[0.0, 17.3]	18.5<br>[6.9, 34.7]	2.1<br>[-1.6, 7.2]	7.4<br>[2.4, 14.0]
	60	f					7.7<br>[0.5, 17.9]		1.5<br>[-1.8, 6.0]	7.9<br>[2.6, 14.7]
	240	r							2.7<br>[-1.4, 8.3]	
	240	f								

A4c. Percentage time above 90°, n_s = 20. Within-day variance, σ^2_{WD}

		t_{tot}	60	60	120	120	240	240	480	480
		n_d	2	4	2	4	2	4	2	4
t_b	1	r	-0.2 [-43.2, 45.9]	0.0 [-40.4, 43.5]	0.5 [-35.6, 39.1]	0.2 [-33.1, 34.9]	0.3 [-31.9, 34.2]	0.2 [-28.7, 29.7]	-0.3 [-29.8, 31.4]	-0.1 [-27.2, 27.1]
	1	f	-0.8 [-41.5, 42.5]	0.8 [-39.0, 43.7]	0.6 [-34.3, 37.3]	0.4 [-32.2, 33.4]	0.8 [-31.3, 33.6]	0.9 [-27.4, 30.5]	1.4 [-29.9, 33.6]	0.3 [-26.2, 25.9]
	15	r	-18.9 [-73.5, 42.1]	-43.3 [-85.3, 4.3]	-6.9 [-53.8, 43.5]	-18.7 [-60.0, 26.1]	-2.0 [-40.3, 38.1]	-7.1 [-41.8, 31.5]	0.6 [-31.6, 34.4]	-1.9 [-31.5, 29.5]
	15	f	-16.3 [-70.4, 42.4]		-9.2 [-55.3, 39.9]	-17.6 [-59.3, 26.5]	-6.0 [-42.7, 32.8]	-9.7 [-43.3, 27.2]	-1.5 [-31.0, 29.2]	-5.9 [-33.8, 23.6]
	60	r			-13.3 [-61.0, 39.5]		-2.9 [-45.6, 42.3]	-13.4 [-50.7, 27.0]	1.0 [-34.0, 36.9]	-2.7 [-34.9, 32.5]
	60	f					3.6 [-38.5, 50.3]		-0.5 [-32.0, 34.0]	4.1 [-29.0, 39.7]
	240	r							2.3 [-33.3, 39.3]	
	240	f								

A5a. Percentage time below 15°, n_s = 10. Between-subjects variance, σ^2_{BS}

		t_{tot}	60	60	120	120	240	240	480	480
		n_d	2	4	2	4	2	4	2	4
t_b	1	r	-7.1 [-139.8,252.6]	-8.6 [-128.6,188.4]	-10.8 [-136.7,240.9]	-8.8 [-122.8,181.6]	-6.2 [-137.2,261.2]	-7.0 [-124.2,184.8]	-4.0 [-136.3,261.9]	-8.3 [-122.3,177.3]
	1	f	-4.6 [-137.8,259.6]	-8.6 [-127.9,181.2]	-6.2 [-133.6,253.5]	-5.0 [-123.6,195.4]	-6.4 [-136.1,254.6]	-6.2 [-121.4,194.4]	-11.4 [-136.8,249.0]	-8.5 [-121.9,181.1]
	15	r	-10.5 [-153.1,269.7]	-7.3 [-153.1,247.3]	-8.0 [-153.1,273.2]	-9.0 [-136.5,206.6]	-9.3 [-149.8,254.8]	-8.9 [-129.3,191.3]	-9.6 [-141.5,253.3]	-7.3 [-124.9,191.5]
	15	f	-7.0 [-153.1,270.9]		-5.7 [-148.8,259.7]	-8.6 [-135.8,203.0]	-4.4 [-145.8,263.5]	-5.3 [-128.3,192.8]	-8.3 [-138.3,265.6]	-2.8 [-124.1,200.9]
	60	r			-8.4 [-153.1,282.6]		-10.0 [-153.1,262.9]	-9.4 [-138.5,212.2]	-10.3 [-147.1,263.1]	-7.9 [-126.9,192.0]
	60	f					-9.6 [-153.1,252.5]		-10.4 [-144.6,272.3]	-7.4 [-127.6,186.3]
	240	r							-14.8 [-152.6,264.3]	
	240	f								

A5b. Percentage time below 15°, n_s = 10. Between-days variance, σ^2_{BD}

		t_{tot}	60	60	120	120	240	240	480	480
		n_d	2	4	2	4	2	4	2	4
t_b	1	r	0.4 [-56.0, 81.2]	0.6 [-46.6, 63.6]	0.3 [-50.0, 80.0]	-0.1 [-39.4, 54.4]	0.6 [-47.5, 79.4]	0.3 [-34.9, 49.3]	1.4 [-46.0, 76.0]	-0.3 [-34.0, 46.1]
	1	f	-5.3 [-58.7, 76.6]	-7.0 [-50.3, 51.0]	-3.7 [-51.5, 73.5]	-5.6 [-42.7, 45.6]	-0.8 [-47.2, 74.5]	-3.3 [-37.9, 45.6]	0.5 [-46.2, 76.1]	-1.8 [-35.6, 44.3]
	15	r	71.7 [-28.0, 217.7]	156.3 [45.8, 297.0]	31.0 [-38.7, 133.8]	73.3 [3.5, 165.7]	12.9 [-42.2, 103.3]	32.4 [-15.8, 100.8]	4.8 [-44.6, 80.8]	12.4 [-27.0, 69.2]
	15	f	59.6 [-33.2, 194.3]		28.4 [-40.2, 131.2]	59.1 [-3.0, 137.5]	12.6 [-42.0, 100.4]	29.9 [-18.1, 93.5]	4.7 [-44.7, 87.6]	10.9 [-26.6, 62.0]
	60	r			61.3 [-28.9, 207.3]		25.2 [-39.5, 134.2]	63.4 [-3.9, 156.9]	8.3 [-42.7, 92.3]	25.7 [-21.6, 95.1]
	60	f					16.4 [-41.6, 106.7]		12.7 [-45.7, 104.5]	16.0 [-24.8, 70.1]
	240	r							21.9 [-41.1, 141.5]	
	240	f								

A5c. Percentage time below 15°, n_s = 10. Within-day variance, σ^2_{WD}

		t_{tot}	60	60	120	120	240	240	480	480
		n_d	2	4	2	4	2	4	2	4
t_b	1	r	0.1<br>[-111.1,111.3]	-0.7<br>[-104.1,102.4]	0.4<br>[-104.3,109.4]	-0.2<br>[-93.3, 98.2]	-0.6<br>[-101.8,101.1]	-1.2<br>[-91.9, 88.5]	0.2<br>[-101.4, 99.6]	-0.9<br>[-89, 91.4]
	1	f	6.9<br>[-104.6,125.1]	8.1<br>[-93.1, 116]	8.8<br>[-95.7, 117]	7.4<br>[-88.7, 104.1]	4.5<br>[-98.4, 105.7]	5.7<br>[-84.5, 97.2]	3.7<br>[-95.6, 102.6]	3.7<br>[-85.5, 94.5]
	15	r	-71.8<br>[-190, 52.1]	-155.8<br>[-250.6, -57.5]	-32.0<br>[-140.2, 80.5]	-69.7<br>[-164.5, 29.1]	-10.3<br>[-116.7, 98.7]	-31.0<br>[-122.7, 63.8]	-4.1<br>[-102.4, 97.4]	-13.1<br>[-99.7, 75.4]
	15	f	-61.1<br>[-182.6, 64.4]		-28.9<br>[-138.2, 88.9]	-61.7<br>[-160.3, 37.6]	-11.8<br>[-115.4, 94.2]	-30.0<br>[-122.4, 62.5]	-0.4<br>[-106.8,104.4]	-10.2<br>[-99.2, 82]
	60	r			-58.1<br>[-163.2, 50.7]		-19.6<br>[-123.7, 87.1]	-58.7<br>[-149.7, 34.9]	-5.8<br>[-106.6, 97.7]	-20.5<br>[-109.1, 72.9]
	60	f					-19.4<br>[-120.2, 81]		-7.2<br>[-109.2, 98.1]	-18.0<br>[-104.8, 72.8]
	240	r							-7.3<br>[-108.1, 92.6]	
	240	f								

A6a. Percentage time below 15°, n_s = 20. Between-subjects variance, σ^2_{BS}

		t_{tot}	60	60	120	120	240	240	480	480
		n_d	2	4	2	4	2	4	2	4
t_b	1	r	-5.8 [-113.8,157.5]	-7.0 [-105.1,123.7]	-8.2 [-109.5,155.3]	-7.8 [-103.1,125.8]	-8.4 [-109.9,149.5]	-8.2 [-103.0,123.4]	-9.1 [-109.6,150.0]	-5.9 [-100.9,123.8]
	1	f	-6.5 [-108.5,155.1]	-8.1 [-103.7,123.2]	-5.9 [-107.8,154.1]	-8.0 [-102.3,119.9]	-6.8 [-107.8,154.9]	-8.3 [-100.3,119.0]	-8.6 [-108.3,144.5]	-5.0 [-100.5,124.5]
	15	r	-6.8 [-153.1,181.3]	-11.6 [-126.3,147.4]	-8.9 [-121.5,165.1]	-9.9 [-114.0,136.3]	-10.0 [-114.6,148.2]	-6.8 [-105.7,130.9]	-7.3 [-111.7,156.1]	-7.8 [-103.5,125.0]
	15	f	-8.9 [-147.3,177.1]		-5.2 [-117.1,164.7]	-5.3 [-110.3,137.7]	-6.2 [-113.8,158.0]	-5.6 [-104.7,134.9]	-9.3 [-109.7,150.3]	-4.4 [-102.6,135.4]
	60	r			-10.6 [-139.1,170.0]		-9.5 [-124.5,163.0]	-10.1 [-111.2,139.3]	-8.7 [-112.6,157.1]	-9.5 [-105.4,123.3]
	60	f					-10.3 [-119.9,150.6]		-10.6 [-114.3,154.2]	-8.4 [-105.7,121.4]
	240	r							-17.2 [-119.8 146.3]	
	240	f								

A6b. Percentage time below 15°, n_s = 20. Between-days variance, σ^2_{BD}

		t_{tot}	60	60	120	120	240	240	480	480
		n_d	2	4	2	4	2	4	2	4
t_b	1	r	0.1 [-43.2, 58.3]	0.0 [-34.6, 43.4]	0.2 [-38.4, 53.8]	0.1 [-29.5, 36.0]	0.4 [-36.4, 50.3]	0.0 [-27.3, 33.4]	0.2 [-35.8, 48.1]	0.3 [-26.7, 32.9]
	1	f	-6.6 [-46.7, 47.2]	-7.0 [-39.1, 33.7]	-3.1 [-41.0, 47.3]	-5.9 [-33.7, 27.2]	-0.4 [-37.3, 50.4]	-3.1 [-29.6, 28.8]	-0.5 [-36.7, 48.8]	-0.7 [-27.0, 31.1]
	15	r	73.1 [-4.1, 176.3]	156.7 [75.0, 254.3]	32.4 [-22.3, 108.8]	71.7 [20.0, 137.8]	14.2 [-29.0, 73.8]	32.2 [-5.0, 78.6]	4.3 [-33.6, 56.6]	13.2 [-17.2, 51.9]
	15	f	59.4 [-12.4, 154.3]		30.9 [-22.7, 101.9]	60.2 [13.9, 114.5]	10.3 [-30.7, 67.6]	29.8 [-5.9, 74.1]	4.5 [-34.5, 60.8]	10.5 [-18.1, 47.1]
	60	r			61.9 [-10.8, 166.6]		26.4 [-24.9, 100.4]	62.4 [12.5, 123.7]	8.1 [-32.6, 66.1]	25.5 [-10.4, 71.2]
	60	f					15.7 [-28.6, 74.4]		13.0 [-33.4, 74.3]	15.3 [-15.7, 51.6]
	240	r							20.8 [-28.8, 99.4]	
	240	f								

A6c. Percentage time below 15°, n_s = 20. Within-day variance, σ^2_{WD}

		t_{tot}	60	60	120	120	240	240	480	480
		n_d	2	4	2	4	2	4	2	4
t_b	1	r	0.4 [-79.5, 81.1]	1.2 [-72.0, 74.8]	-0.1 [-74.9, 76.9]	0.6 [-65.7, 67.9]	0.0 [-69.9, 71.8]	-1.5 [-66.0, 62.5]	0.0 [-71.0, 70.8]	-0.8 [-65.2, 61.4]
	1	f	7.3 [-70.3, 87.2]	7.4 [-66.2, 80.3]	5.7 [-67.8, 79.8]	7.8 [-61.9, 77.5]	4.7 [-66.9, 78.0]	6.2 [-60.6, 72.7]	3.6 [-67.6, 77.0]	4.6 [-60.5, 68.6]
	15	r	-71.1 [-154.8, 14.9]	-154.8 [-219.8,-85.6]	-31.2 [-108.6, 47.7]	-71.2 [-138.8, -3.2]	-11.6 [-84.0, 63.8]	-31.7 [-97.5, 34.7]	-4.3 [-74.8, 67.1]	-10.7 [-73.0, 52.8]
	15	f	-62.2 [-146.6, 27.9]		-28.9 [-106.9, 50.3]	-61.8 [-131.8, 11.9]	-11.5 [-84.4, 64.9]	-29.9 [-95.7, 34.9]	-0.8 [-72.7, 71.1]	-11.9 [-76.2, 53.8]
	60	r			-56.5 [-133.1, 22.7]		-21.1 [-96.7, 55.1]	-56.7 [-120.2, 6.8]	-4.5 [-75.4, 66.4]	-20.7 [-83.7, 44.0]
	60	f					-18.7 [-93.4, 58.4]		-8.2 [-80.4, 66.3]	-19.5 [-83.4, 43.3]
	240	r							-4.8 [-76.4, 67.7]	
	240	f								
